# A structural MRI study of global developmental delay in infants (<2 years old)

**DOI:** 10.3389/fneur.2022.952405

**Published:** 2022-08-18

**Authors:** Hui-miao Sun, Qian-yun Li, Ru-yi Xiao, Ze-dong Zhang, Xiao-yan Yang, Jie Yang, Bo Jin, Jia-xiang Wen, Yan-jun Wu, Hong Yang, Fan Wang

**Affiliations:** ^1^Department of Magnetic Resonance Imaging (MRI), Children Hospital of Shanxi Province (Shanxi Maternal and Child Health Hospital), Taiyuan, China; ^2^College of Medical Imaging, Shanxi Medical University, Taiyuan, China; ^3^Key Laboratory of Biomedical Information Engineering of Ministry of Education, Xi'an Jiaotong University, Xi'an, China

**Keywords:** global developmental delay, infant, cortical surface, CT, SA, MRI

## Abstract

**Objective:**

To use structural magnetic resonance imaging (3D-MRI) to evaluate the abnormal development of the cerebral cortex in infants with global developmental delay (GDD).

**Methods:**

The GDD group includes 67 infants aged between 112 and 699 days with global developmental delay and who underwent T1-weighted MRI scans in Shanxi Children's Hospital from December 2019 to March 2022. The healthy control (HC) group includes 135 normal developing infants aged between 88 and 725 days in Shanxi Children's Hospital from September 2020 to August 2021. Whole-brain T1-weighted MRI scans were carried out with a 3.0-T magnetic resonance scanner, which was later processed using InfantSurfer to perform MR image processing and cortical surface reconstruction. Two morphological features of the cortical surface of the 68 brain regions were computed, i.e., the cortical thickness (CT) and cortical surface area (SA), and compared between the GDD and HC groups.

**Results:**

With regard to the CT, the HC group showed a rapid decrease at first and then a slow increase after birth, and the CT of the GDD group decreased slowly and then became relatively stable. The GDD group showed bilaterally higher hemispherical average CT than those in the HC group. In detail, for the left hemisphere, except in the entorhinal and temporal poles in which the average CT values of the two brain regions were lower than those of the HC group, the CT of the 26 brain regions in the GDD group was higher than those of the HC group (*p* < 0.05). For the right hemisphere, the CT of the entorhinal in the GDD group was lower than that in the HC group. Otherwise, the CT of the remaining 28 brain regions was higher than those in the HC group (*p* < 0.05). With regard to the SA, both groups showed a rapid increase after birth till 23 months and remained quite stable afterward. The GDD group shows lower SA bilaterally than that in the HC group. In detail, SA in the GDD group was lower in most cortical regions of both hemispheres than in the HC group (*p* < 0.05), except for the right temporal pole and entorhinal. When testing for brain asymmetry, we found that the HC group showed obvious asymmetry of CT and SA, while only a few cortical regions in the GDD group showed asymmetry.

## Introduction

The diagnostic criteria of the 5th edition of the Diagnostic and Statistical Manual of Mental Disorders (DSM-V) issued by the American Psychiatric Association on 18 May 18 2013 classify global developmental delay (GDD) as a neurodevelopmental disorder, which refers to underdevelopment in more than two aspects, including skills like motor, language, cognitive and social communication, and adjusts the diagnostic age to <5 years old (1). The prevalence of GDD is around 3%, and 5–10% of healthy children experience GDD early in development. Most cases often have several causes, which are mutually transformed and causative. There is a study that has shown that GDD is associated with genetic defects (2), and Li et al. show the subtle structural changes of each brain area in children with GDD by the change of ADC value (3) to indirectly understand the location and degree of brain injury in children. In a functional imaging study, the UF and SCP WMT showed microstructural changes suggestive of compromised white matter maturation in children with GDD (4). Current international scientific research and clinical evaluation of GDD is mostly based on the Gesell development diagnosis scale, which evaluates the development in five aspects, such as gross movement, fine movement, speech, human ability, and response-ability. The infant is diagnosed with GDD when the development quotient (DQ) is lower than 70 in two or more aspects. The current research regards the age of 0–3 as the key period for the early identification of infants with GDD. Lack of early diagnosis and intervention may introduce further intellectual disability, such as cerebral palsy, autism spectrum disorder (ASD), or attention deficit hyperactivity disorder (ADHD) (5). In particular, the development of language is combined with cognition, which is proceeded for up to 21 months. After 3 years old, cognitive and language development training becomes more difficult, recovery is slow, and the possibility of curing children with GDD is significantly reduced (6). The diagnosis and imaging study of the GDD is of great significance to the choice of treatment, prognosis, risk assessment of recurrence, and the implementation of prevention programs. At present, few studies are focusing on GDD with whole-brain structure MR images.

The first 2 years of life are a period of abnormal dynamic development of the structure and functions of the human brain. Babies' brains reach 80% of their adult size at the age of 2 (7). Studies have shown that many neurodevelopmental and mental disorders are caused by abnormal brain development at this stage. The cerebral cortex, which makes up the largest part of the human brain, has the topology of a 2-D sheet and a highly folded geometry (8). Surface-based morphometry (SBM) is widely used and mature in estimating cortical morphological indexes such as volume, cortical thickness, and surface area. Cortical thickness (CT) and surface area (SA) are important components that measure cortical morphometry. CT and SA abnormalities are commonly observed in neurodevelopmental disorders, including bipolar disorder (9), schizophrenia (10), autism (11), and attention-deficit/hyperactivity disorder (12). Shaw et al. used 3D-T1WI's longitudinal study of the correlation between intelligence and cortical thickness in normal children and adolescents and found that in early childhood, there was a significant negative correlation between intelligence and cortical thickness, while with age, they gradually showed a positive correlation (13). Therefore, the selection of these two indicators for quantitative analysis of children with GDD is helpful to further explore the relevant pathophysiological mechanism.

An MRI can associate the development of the brain structure with the behavior of infants, making it convenient to evaluate the lesions. In particular, 3D-MRI uses three-dimensional volume scanning with high spatial resolution and good tissue contrast and can display brain structure at the submillimeter level, which is convenient for the establishment of a visual map of the human brain. Infant FreeSurfer (14) is the most advanced special cortex analysis software for infants, which can calculate the morphological parameters of any position of the brain or other related data. The purpose of this study is to quantitatively analyze the CT and SA of infants with GDD by using whole-brain 3D-MRI, draw the developmental trajectory maps, and analyze the hemispheric asymmetry to help find the brain structural changes related to the disease and further reveal the potential pathophysiological mechanism of GDD.

Global developmental delay is a temporary diagnosis, which can be returned to normal after a timely clinical intervention. The use of structural magnetic resonance imaging (3D-MRI) to evaluate the abnormal development of the cerebral cortex in children with global developmental delay is conducive to providing early imaging evidence for the clinic and analyzing the differences in different brain regions, providing support for the study of the mechanism of related neuropsychiatric disorders.

## Data and methods

### General information

The experimental group selected 67 infants who underwent 3D-T1WI MRI examination in Shanxi Children's Hospital from December 2019 to March 2022 as the GDD group. They were aged between 112 and 699 d and included the following criteria: (1) met the diagnostic criteria of GDD in DSM-V; (2) the age was between 0 and 2 years old; (3) there was no previous neurotrophic factor drug therapy; (4) the image quality was good, and accurate data could be obtained. Exclusion criteria: infantile schizophrenia, obsessive-compulsive disorder, autism spectrum disorder, Asperger's syndrome, and other diseases. In the HC group, 145 infants were selected who underwent T1-weighted brain MRI examination in Shanxi Children's Hospital from September 2020 to August 2021, aged from 88 to 725 days. Inclusion criteria were as follows: (1) full-term natural delivery; (2) no family history of mental or neurological disease; (3) no intracranial space occupying or congenital disease by clinical and imaging examination; (4) normal motor and cognitive function tested by development scale. All children complete the examination under sedation. This study was approved by the ethics committee of Shanxi Children's Hospital.

### Inspection method

For the 3D-T1WI data collection, all subjects were given an enema with 5% chloral hydrate, a dose of 1 ml/kg, equipped with a hearing protection device, and scanned after deep sleep.

The GDD group was examined with GEDISCOVERY MR750W3.0T magnetic resonance machine and the head matrix coil. Sweep parameters were as follows: (1) regular MRI: T1WI: TR = 1,750 ms, TE = 27 ms; T2WI: TR = 5,231 ms, TE = 129 ms; T2-FLAIR: TR = 7,800 ms, TE = 89 ms. *All sequences*: FOV = 200 × 200 mm^2^, matrix = 256 × 256 mm^2^, slice spacing = 1.2 mm, slice thickness = 5.0 mm, excitation times = 2. (2) 3D-T1WI sequence: TR = 7.7 ms, TE = 2.8 ms, FOV = 240 × 240 mm^2^, matrix = 256 × 256 mm^2^, slice spacing = 0 mm, slice thickness = 1.0 mm, excitation times = 1.

The HC group was examined with PhilipsAchieva3.0T magnetic resonance machine and the head matrix coil. MR scan sequences include: (1) 3D-T1WI: using gradient echo sequence, TR = 600 ms, TE = 27 ms, FOV = 250 × 250 mm^2^, slice spacing = −0.55 mm, slice thickness = 1.1 mm. (2) T2WI: TR = 2,651 ms, TE = 105 ms, FOV = 180 × 180 mm^2^, matrix = 0.9 × 0.9 mm^2^, slice spacing = 0.5 mm, slice thickness = 4.0 mm, excitation times = 2. (3) T2-FLAIR: TR = 7,800 ms, TE = 89 ms, TI = 2,300 ms, FOV = 180 × 180 mm^2^, matrix = 0.9 × 0.9 mm^2^, slice spacing = 0.5 mm, slice thickness = 4.0 mm, excitation times = 2.

### Data processing

In this experiment, the Infant FreeSurfer software (11) was used to reconstruct the three-dimensional cortical surface of all 3D-T1WI magnetic resonance image data, including image intensity correction, head stripping, brain tissue segmentation, left and right cerebral dissection, reconstruction of the inner and outer surface of the cerebral cortex, and so on. To ensure the quality of skull dissection and the accuracy of gray matter/white matter boundary segmentation, the results of skull dissection and brain tissue segmentation were examined by two skilled anatomical operators. After the cortical reconstruction was completed, the left and right hemispheres were divided into 33 brain regions according to the FreeSurfer cortical atlas (15), and the average CT and the summed SA of each brain region were calculated. Linear mixed effect (LME) (16) models were used to model the development trajectory, and three models (linear, quadratic, and logistic curve) were used to fit the trajectory. After fitting different models, the best model was selected as the development trajectory according to the Akaike Information Criterion (AIC). In this study, based on this model, the developmental trajectories of CT and SA of two groups of subjects with age were fitted, respectively.

### Statistical methods

MATLAB software was used to analyze the differences in the CT and SA between the two groups and the asymmetry between the left and right hemispheres of the two groups. Double-sample unpaired *t*-test was used for the difference between the groups, and paired *t*-test was used for hemispheric asymmetry. *P* < 0.05 was statistically significant.

## Results

### Developmental trajectories of CT

**Figure 1:**
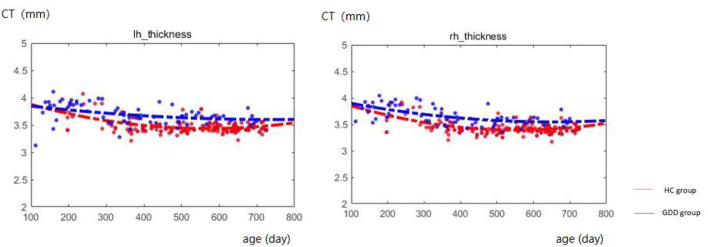
Hemispherical CT developmental trajectories of the two groups. The red line represents the HC group, and the blue line represents the GDD group (lh, left hemisphere; rh, right hemisphere).

**Figure 2:**
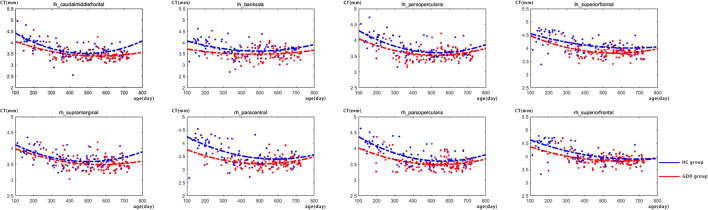
There are the CT developmental trajectories of some representative regions of the two groups. The red line represents the HC group, and the blue line represents the GDD group (lh, left hemisphere; rh, right hemisphere).

Figure 1 shows the comparative maps of the CT developmental trajectory of bilateral cerebral hemispheres between the two groups. Figure 2 shows the CT developmental trajectories of some representative regions of the two groups. Red represents the HC group and the blue line represents the GDD group. Different from the trend of rapid decrease and then slow increase of average CT in the HC group, the average CT of the GDD group decreases slowly and then remains stable. From the developmental trajectory maps, it can be seen that the average CT values of both sides of the brain in the GDD group are higher than those in the HC group.

### Developmental trajectories of SA

**Figure 3:**
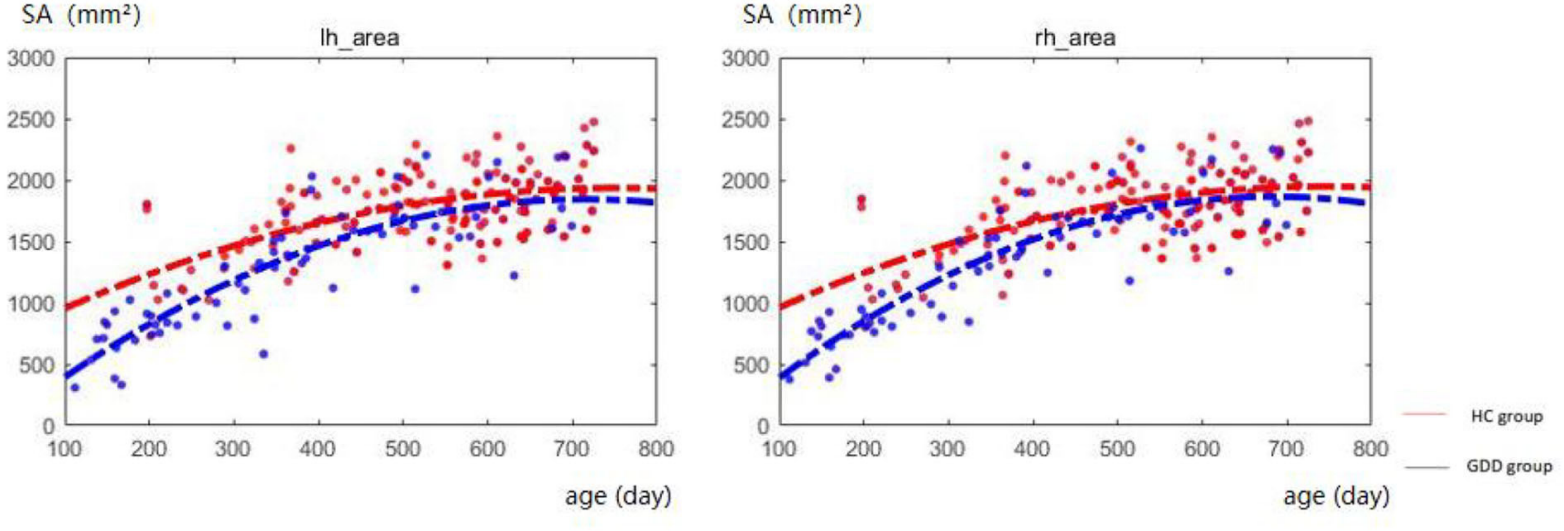
Hemispherical SA developmental trajectory between the two groups. The red line represents the HC group, and the blue line represents the GDD group (lh, left hemisphere; rh, right hemisphere).

**Figure 4:**
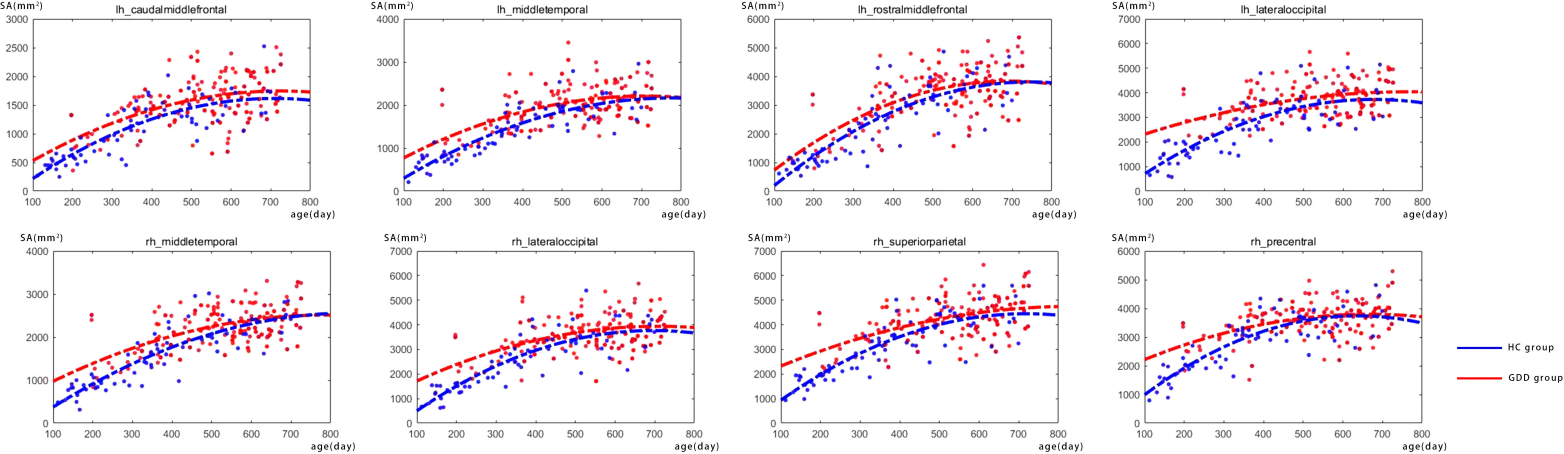
There are the SA developmental trajectories of some representative regions of the two groups. The red line represents the HC group, and the blue line represents the GDD group (lh, left hemisphere; rh, right hemisphere).

Figure 3 shows the comparative maps of the hemispherical SA developmental trajectory between the two groups. Figure 4 shows the SA developmental trajectories of some representative regions of the two groups. Red represents the HC group and the blue line represents the GDD group. The average SA of both groups increases rapidly at first, reaches the peak at about 23 months, and then remains stable. From the developmental trajectory maps, it can be seen that the average SA value of both sides of the brain in the GDD group is lower than that in the HC group.

### Differences in CT between two groups

Table 1 shows brain regions with significant differences in cortical thickness (CT) of bilateral cerebral hemispheres between the two groups. As shown in Table 1, for the left cerebral hemisphere, except for the average CT values of the entorhinal and temporal pole are lower than that of the HC group, the CT values of 26 brain regions such as caudal middle frontal, postcentral, pars-triangularis, supra-marginal, and bankssts in the GDD group are higher than those of the HC group, while for the right cerebral hemisphere, except that the CT value of entorhinal is lower than that of the HC group, the CT values of 28 brain regions such as superior temporal, posterior cingulate, inferior parietal, precentral and transverse temporal in the GDD group are higher than those of the HC group.

**Table 1:** Brain cortical regions that show significant differences in cortical thickness (CT) between the two groups.

**Brain region**	**CT (mm)**		***P***	
	**Left**	**Right**	**Left**	**Right**
	**hemisphere**	**hemisphere**	
	**(GDD/HC)**	**(GDD/HC)**	
Caudal middle frontal	3.76/3.46	3.80/3.44	<0.0001**	<0.0001**
Entorhinal	2.27/3.08	2.56/3.07	<0.0001**	0.0005**
Postcentral	3.20/2.83	3.20/2.81	<0.0001**	<0.0001**
Pars triangularis	3.89/3.68	3.93/3.60	<0.0001**	<0.0001**
Supra marginal	3.81/3.63	3.73/3.54	<0.0001**	<0.0001**
Bankssts	3.74/3.51	3.85/3.58	<0.0001**	<0.0001**
Lateral orbitofrontal	4.11/4.12	3.84/3.92	0.9246	0.0464*
Pars orbitalis	4.22/4.03	4.02/3.90	<0.0001**	0.0112*
Middle temporal	4.05/3.74	4.09/3.78	<0.0001**	<0.0001**
Pericalcarine	3.43/2.93	3.31/2.92	<0.0001**	<0.0001**
Paracentral	3.61/3.22	3.66/3.27	<0.0001**	<0.0001**
Medial orbitofrontal	4.03/4.05	4.09/3.99	0.799	0.0406*
Frontalpole	4.51/4.37	4.52/4.38	0.0446*	0.0037**
Cuneus	3.82/3.25	3.71/3.23	<0.0001**	<0.0001**
Inferior temporal	4.04/3.68	4.05/3.69	<0.0001**	<0.0001**
Rostral middle frontal	4.12/3.74	4.08/3.70	<0.0001**	<0.0001**
Isthmus cingulate	2.91/2.66	2.86/2.62	<0.0001**	<0.0001**
Lateral occipital	3.86/3.16	3.81/3.16	<0.0001**	<0.0001**
Lingual	3.57/3.24	3.51/3.23	<0.0001**	<0.0001**
Superior parietal	3.73/3.26	3.70/3.23	<0.0001**	<0.0001**
Pars opercularis	3.80/3.58	3.83/3.54	<0.0001**	<0.0001**
Fusiform	3.86/3.53	3.72/3.49	<0.0001**	<0.0001**
Superior frontal	4.19/3.88	4.16/3.88	<0.0001**	<0.0001**
Temporalpole	3.86/4.09	3.85/3.95	0.0018**	0.1032
Precuneus	3.92/3.52	3.81/3.51	<0.0001**	<0.0001**
Transverse temporal	3.26/3.09	3.31/3.15	<0.0001**	0.0002**
Precentral	3.36/3.06	3.32/3.01	<0.0001**	<0.0001**
Inferior parietal	3.92/3.64	3.90/3.59	<0.0001**	<0.0001**
Posterior cingulate	3.37/3.13	3.17/2.91	<0.0001**	<0.0001**
Superior temporal	3.70/3.49	3.77/3.46	<0.0001**	<0.0001**
Insula	3.73/3.68	3.76/3.72	0.14	0.2719
Rostral anterior cingulate	3.29/3.40	3.11/3.09	0.1156	0.6732
Caudal anterior cingulate	3.05/3.03	2.99/2.92	0.7688	0.2132

*P <0.05, **P <0.01.

### Differences in SA between two groups

Table 2 shows brain regions with significant differences in cortical surface areas (SA) of bilateral cerebral hemispheres between the two groups. As shown in Table 2, for the left hemisphere, SA values in all 33 brain regions in the GDD group are lower than those in the HC group; for the right hemisphere, SA values in 31 brain regions in the GDD group are also lower than those in the HC group, except the entorhinal and temporal pole.

**Table 2:** Brain cortical regions that show significant differences in cortical surface area (SA) between the two groups.

**Brain region**	**SA (mm** ^**2**^ **)**		***P***	
	**Left**	**Right**	**Left**	**Right**
	**hemisphere**	**hemisphere**	
	**(GDD/HC)**	**(GDD/HC)**	
Caudal middle frontal	1087.77/1549.43	1132.23/1448.11	<0.0001**	<0.0001**
Entorhinal	123.46/164.58	172.18/151.78	0.0076**	0.5221
Postcentral	2713.43/3385.09	2839.90/3212.46	<0.0001**	<0.0001**
Pars triangularis	619.24/856.49	849.88/1022.06	<0.0001**	<0.0001**
Supra marginal	2073.40/2682.30	2191.27/2560.47	<0.0001**	<0.0001**
Insula	1376.64/1669.19	1336.84/1566.32	<0.0001**	<0.0001**
Bankssts	509.30/719.18	491.03/676.99	<0.0001**	<0.0001**
Lateral orbitofrontal	1095.04/1384.28	1373.65/1475.09	<0.0001**	<0.0001**
Pars orbitalis	262.99/375.67	400.81/466.79	<0.0001**	<0.0001**
Middle temporal	1389.69/1991.66	1728.52/2211.28	<0.0001**	<0.0001**
Pericalcarine	640.50/971.46	786.29/1076.84	<0.0001**	<0.0001**
Paracentral	842.28/1021.16	1014.06/1128.39	<0.0001**	<0.0001**
Medial orbitofrontal	873.94/1048.12	943.83/1094.34	<0.0001**	<0.0001**
Frontalpole	120.06/158.83	190.28/206.51	<0.0001**	<0.0001**
Cuneus	721.13/1079.72	915.14/1137.95	<0.0001**	<0.0001**
Inferior temporal	1355.59/1951.14	1485.49/1877.82	<0.0001**	<0.0001**
Rostral middle frontal	2368.85/3392.16	2823.70/3521.96	<0.0001**	<0.0001**
Rostral anterior cingulate	369.22/498.77	370.93/464.99	<0.0001**	<0.0001**
Isthmus cingulate	751.63/1056.92	802.35/1008.20	<0.0001**	<0.0001**
Lateral occipital	2644.76/3719.88	2846.88/3600.93	<0.0001**	<0.0001**
Lingual	1409.71/2100.67	1695.88/2121.28	<0.0001**	<0.0001**
Superior parietal	3070.07/4349.31	3429.06/4196.36	<0.0001**	<0.0001**
Pars opercularis	782.91/1094.37	775.27/968.42	<0.0001**	<0.0001**
Fusiform	1296.44/2002.08	1465.54/1932.85	<0.0001**	<0.0001**
Caudal anterior cingulate	373.16/464.14	479.48/570.34	<0.0001**	<0.0001**
Superior frontal	3445.20/4450.62	3795.94/4287.15	<0.0001**	<0.0001**
Temporal pole	251.08/297.65	308.28/266.77	0.0026**	0.9663
Precuneus	1900.47/2825.29	2326.39/3033.29	<0.0001**	<0.0001**
Transverse temporal	294.05/372.20	241.71/294.85	<0.0001**	<0.0001**
Precentral	2840.55/3590.41	3055.08/3590.00	<0.0001**	<0.0001**
Inferior parietal	2187.09/3243.82	2868.38/3747.84	<0.0001**	<0.0001**
Posterior cingulate	712.06/928.79	790.42/957.84	<0.0001**	<0.0001**
Superior temporal	2155.62/2789.43	2304.05/2754.76	<0.0001**	<0.0001**

**P <0.01.

### Asymmetry of CT and SA of the two groups

**Figure 5:**
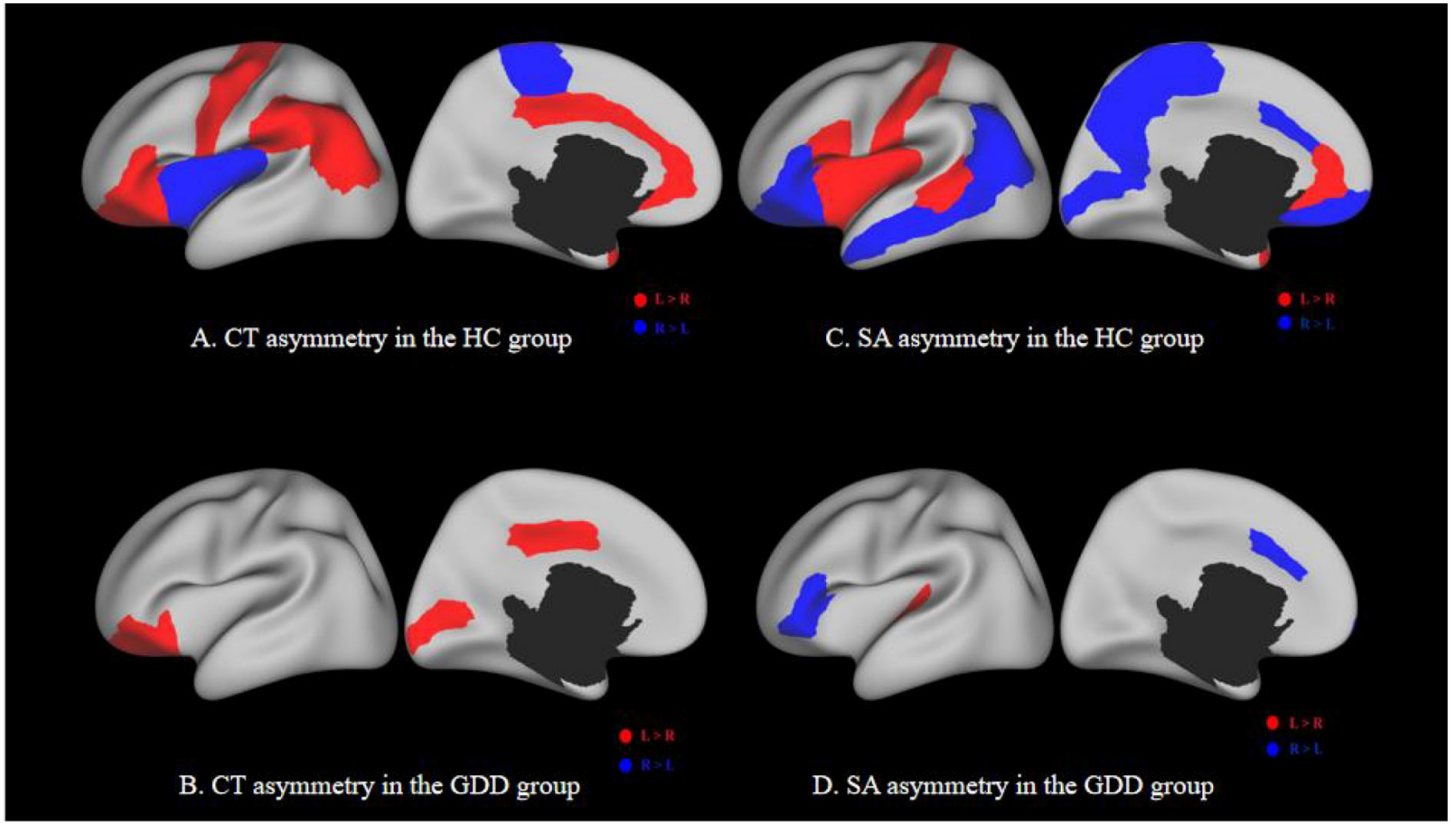
**(A,C)** show the asymmetry of CT and SA between two hemispheres of the HC group. **(B,D)** show the asymmetry of CT and SA between two hemispheres of the GDD group. Each image has a lateral view on the left and a medial view on the right. Red indicates a higher CT/SA in the left hemisphere of the same region than the right, while blue indicates the opposite. The black hole in the medial view indicates the medial wall between two hemispheres.

The medial and lateral views of Figures 5A,C show the asymmetry of CT and SA between the left and right hemispheres of the HC group. The medial and lateral views of Figures 5B,D show the asymmetry of CT and SA between the left and right hemispheres of the GDD group. All results are shown on the average central cortical surface of the age-matched left hemisphere. On the medial and lateral surfaces, the overall patterns in the left greater than the right (red), and the right greater than the left (blue) are relatively consistent in all ages.

As shown in Figure 5B and Table 3, the significantly asymmetric brain regions of CT in the GDD group are the lateral orbitofrontal, pars-orbitalis, pericalcarine, and posterior cingulate, all of which are larger on the left side than on the right side.

**Table 3:** Brain cortical regions that show significant asymmetry in cortical thickness (CT) in the GDD group.

**Brain hemisphere**	**CT (mm)**		***P***
	**Left hemisphere**	**Right hemisphere**	
Lateral orbitofrontal	4.13	3.87	0.0017**
Pars orbitalis	4.26	4.08	0.0237*
Pericalcarine	3.46	3.31	0.0489*
Posterior cingulate	3.37	3.18	0.0177*

*P <0.05, **P <0.01.

As shown in Figure 5D and Table 4, the significantly asymmetric brain regions of SA in the GDD group are pars-triangularis, pars-orbitalis, frontal pole, caudal anterior cingulate, and transverse temporal, all of which are larger on the right side than on the left side except transverse temporal.

**Table 4:** Brain cortical regions that show significant asymmetry in cortical surface area (SA) in the GDD group.

**Brain hemisphere**	**SA (mm** ^**2**^ **)**		***P***
	**Left hemisphere**	**Right hemisphere**	
Pars triangularis	604.62	751.4	0.0174*
Pars orbitalis	258.57	349.88	0.0016**
Frontalpole	114.72	163.92	0.0003**
Caudal anterior cingulate	364.02	430.44	0.0327*
Transverse temporal	283.14	208.17	0.0008**

*P <0.05, **P <0.01.

Comparing Figures 5A,C with Figures 5B,D, it can be seen that the asymmetry area of the brain in the GDD group is less than that in the HC group. Except that the CT of the pericalcarine in the GDD group is larger than that on the right side, the asymmetry of other brain regions is consistent with that of the HC group.

## Discussion

As we all know, the development of the cerebral cortex is closely related to the realization of various functions of the human body. For example, the frontal lobe is the area of executive function, attention, and motor coordination; the parietal lobe is involved in the development of spatial orientation, speech and language, and attention; the temporal lobe is associated with memory integration; and the occipital lobe is the visual center (17), while the insular lobe connects the other lobes to participate in the realization of cognitive and sensory functions. Developmental disorders in any part of the cerebral cortex can lead to motor, language, and cognitive disorders.

At present, there are various forms of research on the cerebral cortex, and surface-based morphometry (SBM) is more in line with the goal of this study. We selected two indexes: cortical thickness and cortical surface area. Panizzon and other studies have shown that cortical thickness and cortical surface area are genetically related, but there is no genetic relationship between them (18), which further proves that they are indeed driven by different cellular mechanisms, which is consistent with the results of Rakic (16, 19). There have been similar studies on the properties of normal human brain structural networks, which have proved that the description of surface area and cortical thickness reveal different properties of human brain network structures (20). Grasby et al. showed that genetic factors had opposite effects on surface area and thickness and observed that there was a significant positive genetic correlation and two-way causality between total surface area and general cognitive function and education level, and a significant negative correlation between total surface area and insomnia, attention deficit hyperactivity disorder, depressive symptoms, major depressive disorder, and neuroticism (21). Therefore, this study compared the normal development of infants from the results of statistical differences between the two indicators, respectively, to explore the mechanism of neurodevelopmental disorders related to general developmental delay.

To eliminate the possible research differences caused by different scanning devices, homogenization of the data of different devices was carried out. We collected the image data of nineteen normally developing infants aged between 78 and 940 days who were examined by GEDISCOVERYMR750W3.0T magnetic resonance machine and met the inclusion criteria. The scanning parameters are the same as those of GEDISCOVERYMR750W3.0T magnetic resonance machine in the study. A new control group was formed between the above subjects and the control subjects examined by PhilipsAchieva3.0T magnetic resonance machine in the study. The new control group and the experimental group scanned by GEDISCOVERYMR750W3.0T magnetic resonance machine in the study used the same data processing method to compare the difference between CT and SA in each brain area of the same bilateral brain. Only one of the brain regions on both sides showed different results compared to existing results, but the differences were not significant. In summary, we believe that the influence of different scanning devices on the research results can be ignored.

### GDD and HC show different development trajectories

In this study, the average CT of the bilateral brain in the GDD group was slightly different from that in the HC group, which decreased rapidly after birth and then kept stable. This is different from the results of some literature works. Wang et al. showed that in the first 2 years after birth, the average CT development of the whole cerebral cortex followed an “inverted U-shaped” trajectory, and CT increased dynamically in the first year but changed slightly in the second year (22). It may be related to genetic, dietary, and environmental factors due to the different sources of the subjects. On the other hand, the average SA development trajectory of both sides of the brain in the GDD group was similar to that in the HC group, which increased rapidly at first after birth, reached a peak at about 23 months, and remained quite stable afterward. It is suggested that the SA expansion pattern of children with global developmental delay is similar to that of normal children.

In addition, from the developmental trajectory map, we can see that the average CT value of both hemispheres in the GDD group is higher than that in the HC group, and the average SA value in the bilateral brain in the GDD group is lower than that in the HC group. The relevant content is further analyzed in the following content.

### GDD and HC show differences in CT

This study found that for the left cerebral hemisphere, except for insula, the lateral orbitofrontal cortex, medial orbitofrontal cortex, caudal anterior cingulate, and rostral anterior cingulate, the CT values of other brain regions in the GDD group were different from those in the HC group. The CT values of the entorhinal and temporal pole in the GDD group were lower than those in the HC group, while the CT values in the other frontal, temporal, parietal, and occipital lobes were higher than those in the HC group. For the right cerebral hemisphere, except insula, temporal pole, caudal anterior cingulate, and rostral anterior cingulate, the CT values of other brain regions in the GDD group were different from those in the HC group, in which the entorhinal CT value in the GDD group was lower than that in the HC group, while the CT values in the other frontal, temporal, parietal and occipital lobes were higher than those in the HC group. Since the global developmental delay may evolve into autism spectrum disorder and hyperactive attention deficit to a certain extent, the author studies a similar mechanism.

The results of this study showed that the CT values of superior frontal, caudal middle frontal, pars-opercularis, pars-orbitalis, and posterior cingulate increased in both hemispheres compared with those of the HC group. The lateral orbitofrontal cortex and medial orbitofrontal cortex also showed an increase on the right. In van Rooij's study on autism (23), the same results were observed in the same areas, which may indicate that the children with global developmental delay have the same motor and cognitive control disorder mechanism, resulting in a developmental delay in the corresponding dimension.

In Yang et al.'s study, it was found that the thinning of the right superior frontal gyrus was consistent with the typical symptoms of ADHD. These structural abnormalities may correspond to disorders of attention, executive function, and cognitive control (24). In this study, the increase in CT value of the right superior frontal may indicate that part of the neurodevelopmental disorder mechanism of motor execution and cognition in children with global developmental delay is opposite to that of ADHD. In addition, compared with the results of this study, Kong et al. also found an increase in cortical thickness in the right frontal pole, right medial orbitofrontal gyrus, and right anterior and posterior central gyrus in children with Tourette syndrome (25). The frontal pole and medial orbitofrontal cortex belong to the prefrontal cortex, which is related to the thinking and execution of the brain, while the precentral gyrus and postcentral gyrus belong to the sensorimotor cortex. The increased cortical thickness in these areas may indicate that the neurons in these areas are structurally dense and can increase the ability to regulate convulsions (21). These findings may show a compensatory effect, but it may also be due to the inhibition of exercise and other abilities caused by a too thick CT, which needs further study.

The upper parietal lobe is part of the default mode network. The default mode network has a functional connection to the caudate nucleus through dopamine projection. The striatal dopaminergic circuit may regulate cognition and emotion by regulating this network. In this study, it was found that the CT value of the bilateral parietal lobe was higher than that of the HC group, which may cause cognitive impairment in children with global developmental delay. This is similar to the related results of Zhang et al. on depression (26).

This study found that CT increased and SA decreased in the bilateral temporal lobe and fusiform, which may cause the disturbance of social perception. Adolphs believes that the higher sensory cortex, such as the fusiform gyrus and superior temporal sulcus, is involved in detailed sensory processing (27). Zilbovicius's study of autism spectrum disorders has also shown that the superior temporal sulcus is a major participant in social perception (28). In the study of ADHD, Hoogman et al. found that the surface area of children with ADHD was lower, mainly in the frontal lobe, cingulate gyrus, and temporal lobe, and the thickness of the fusiform gyrus and temporal pole cortex was also lower (29). However, this study is contrary to the results in terms of cortical thickness. Due to the differences in cellular mechanisms between CT and SA, it is not possible to determine which role or the combined effect of the two causes the corresponding dysfunction.

### GDD and HC show differences in SA

The results of this study showed that except for the right entorhinal and the temporal pole, the SA in all brain regions of the other bilateral hemispheres was lower than that of normal children. During the development of normal children, the lateral temporal lobe, lateral parietal lobe, and medial prefrontal lobe were highly dilated, and the medial temporal lobe was poorly dilated. Sensory-specific correlative cortex such as supraoccipital gyrus (visual correlation), superior temporal gyrus/middle gyrus (auditory correlation), and superior parietal gyrus (sensory/tactile correlation/spatial correspondence) also showed a significant increase in SA (30). Fjell et al. showed that several regions in the frontal cortex, especially the anterior cingulate, showed high expansion in both development and evolution (31), in which the area of these regions was related to intellectual functions in humans. The lack of cortical dilatation in children with global developmental delay may indicate developmental disorders in the corresponding brain regions.

In SA-related studies of autism, cortical dilatation occurs in high-risk infants with ASD from June to 12 months, and excessive brain volume growth is associated with the occurrence and severity of social deficits in autism (32). The data of this study show that the SA of multiple brain regions of both hemispheres is lower than that of normal children, indicating that the decreased social ability of children with global developmental delay may be different from the pathophysiological mechanism of ASD expansion in SA. In a study on subjective cognitive decline, it was found that the decrease in total cortical volume and cortical surface area in patients (33) may suggest the pathophysiological mechanism of decreased cortical surface area in children with global developmental delay. Another study on autism spectrum disorders showed a significant decrease in SA in the orbitofrontal cortex and posterior cingulate gyrus (34), which is consistent with the results of this study, suggesting that the decline in executive power may be related to this.

At present, most of the research results on neurodevelopmental disorders are focused on adolescents and adults, and they are different from the results of this study and there are not many references, so the mechanism of SA reduction in children with global developmental delay needs further research.

### GDD and HC show different asymmetry patterns

The asymmetrical areas of the brain in the GDD group were less than those in the HC group. The CT of the GDD group showed significant left deviation in lateral orbitofrontal, pars-orbitalis, peri-calcarine, and posterior cingulate. The SA of the GDD group showed significant left deviation in transverse temporal and significant right deviation in pars-triangularis, pars-orbitalis, frontal pole, and caudal anterior cingulate.

Normal children show obvious left deviation in the early stage of infants (35), but in this study, the number of lateral brain areas in the GDD group is less than that in the HC group, which may be caused by underdevelopment, indicating that there may be maturation disorders in the corresponding brain regions. Asymmetry between the left and right hemispheres is an important aspect of human brain tissue, which may be changed under various neurodevelopmental abnormalities (35).

The left hemisphere responsible for language specialization is one of the earliest observed brain asymmetries. Some aspects of language generation and syntactic processing are then mainly located in the triangle and operculum of the inferior frontal gyrus (36). The results of this study show that the left tilting areas of CT and SA in the whole frontal lobe, including the inferior frontal gyrus, are fewer than those in the HC group, indicating that the decrease in lateralization may lead to the corresponding language development delay. Glasser's neurography analysis using diffusion imaging data showed that the arcuate bundle connecting the superior temporal gyrus (STG) and the middle temporal gyrus (MTG) to the inferior frontal lobe was asymmetrical to the left, and the left STG and MTG pathways were involved in speech processing and lexical-semantic processing, respectively (37). This left deviation was significantly reflected in the CT hemispheric asymmetry of middle temporal in the HC group, but not in the GDD group. It is further proved that the language cortex of the left hemisphere may not be fully activated, which is related to the underdevelopment of language in children.

Li et al. showed the leftward asymmetries in the medial prefrontal, paracentral, and anterior cingulate cortices, which expanded substantially during the first 2 years of normal infants (38). In this study, we have not seen the leftward asymmetries in the same regions. Postema et al. showed that ASD was significantly associated with alterations of cortical thickness asymmetry mostly in the medial frontal, orbitofrontal, cingulate, and inferior temporal areas, and also with asymmetry of the orbitofrontal surface area (39). This study does not show the same results. This may indicate that children with GDD have a disorder in the lateralization of the relevant cortex, but also avoid the risk of ASD to a certain extent.

Some studies have shown that children with attention deficits have consistent functional disorders in the right inferior frontal gyrus and anterior cingulate cortex (40, 41). The results of this study showed that the lateralization of the corresponding areas in the GDD group was normal, and the thickness of the bilateral anterior cingulate cortex was not different from that in the HC group, indicating that the GDD group may not have cortical dysfunction in the corresponding regions.

## Conclusion

The results of this study show that compared with normal developmental children, the whole brain average developmental trajectory of GDD infants is similar to that of HC infants, but there is an increase in CT and a decrease in SA in many brain regions on both sides of the brain. There may be no correlation between the two aspects, and the specific pathophysiological mechanism needs to be further studied.

The results of this study are helpful for further analyzing the mechanism of neurodevelopmental disorders in children with GDD, providing visual imaging data for clinical diagnosis and treatment, prognosis evaluation, efficacy evaluation and correlation analysis with clinical score, and facilitating further research work related to infant brain development.

## Limitation

Because our sample size is limited, there is no grouping according to etiology and backwardness dimensions, so the final result can only be an overall difference. Next, we will expand the sample size, take etiology and other factors into correlation considerations, and study the specific mechanisms of neurodevelopmental disorders in different dimensions of developmental backwardness.

## Data availability statement

The original contributions presented in the study are included in the article/Supplementary material, further inquiries can be directed to the corresponding author/s.

## Ethics statement

Written informed consent was obtained from the individual(s), and minor(s)' legal guardian/next of kin, for the publication of any potentially identifiable images or data included in this article.

## Author contributions

H-mS: project guidance, data collection and analysis, and article writing and modification. Q-yL: project implementation, data collection and analysis, and article writing and revision. RX: data post-processing, statistical analysis, and article modification. Z-dZ and J-xW: clinical data collection. X-yY: interpretation of results. JY, BJ, and Y-jW: image data collection. HY: image examination and scanning. FW: project guidance, data post-processing, and article modification. All authors contributed to the article and approved the submitted version.

## Funding

This work was funded by Key Research and Development Program of Shanxi Province (Project No.: 201903D321051).

## Conflict of interest

The authors declare that the research was conducted in the absence of any commercial or financial relationships that could be construed as a potential conflict of interest.

## Publisher's note

All claims expressed in this article are solely those of the authors and do not necessarily represent those of their affiliated organizations, or those of the publisher, the editors and the reviewers. Any product that may be evaluated in this article, or claim that may be made by its manufacturer, is not guaranteed or endorsed by the publisher.
